# Exploring patient satisfaction after operative and nonoperative treatment for midshaft clavicle fractures: a focus group analysis

**DOI:** 10.1186/s12891-020-03557-y

**Published:** 2020-08-18

**Authors:** Eric D. Tutuhatunewa, Martin Stevens, Olivier C. Dams, Jeffrey van Son, Rebecca D. Louhanepessy, Paul F. M. Krabbe, Maarten J. Postma, Ron L. Diercks

**Affiliations:** 1 Department of Orthopedic Surgery, University Medical Center Groningen, University of Groningen, Groningen, The Netherlands; 2 Department of Epidemiology, University of Groningen, University Medical Center Groningen, Groningen, The Netherlands; 3 Department of Pharmacy, Groningen, Unit of PharmacoTherapy, -Epidemiology & -Economics (PTE2), University of Groningen, Groningen, The Netherlands; 4 Unit of Global Health, Department of Health Sciences, University of Groningen, University Medical Center Groningen, Groningen, The Netherlands; 5 Department of Economics, Econometrics & Finance, University of Groningen, Faculty of Economics & Business, Groningen, The Netherlands

**Keywords:** Patient satisfaction, Focus group, Qualitative research, Patient experience, Clavicle fracture, Shoulder

## Abstract

**Background:**

There is no consensus on the optimal treatment for displaced midshaft clavicle fractures. Several studies indicate superior patient satisfaction in favour of operative reconstruction. It is unknown what drives superior satisfaction in this treatment group. The aim of this study was to explore patient satisfaction and identify contributors to patient satisfaction after operative and nonoperative treatment for displaced midshaft clavicle fractures in adults using a focus group approach.

**Methods:**

Four face-to-face and two web-based focus groups were hosted. A total of 24 participants who were treated nonoperatively (*n* = 14) or operatively (*n* = 10) agreed to participate. Participants were selected using purposive sampling, ensuring variation in gender, age, treatment complications and outcomes. A question script was developed to systematically explore patient expectations, attitudes and satisfaction with different dimensions of care. All focus groups were voice-recorded and transcribed at verbatim. Thematic analysis was conducted on all face-to-face and web-based transcripts.

**Results:**

The main emerging themes across treatment groups were; need for more information, functional recovery, speed of recovery and patient-doctor interaction. There was no difference in themes observed between operative and nonoperative focus groups. The lack of information was the most important complaint in dissatisfied patients.

**Conclusion:**

Our study shows that informing patients about their injury, treatment options and expectations for recovery is paramount for overall patient satisfaction after treatment for a displaced midshaft clavicle fracture.

**Level of evidence:**

Level III, focus group study.

## Introduction

Clavicle fractures are a common injury, representing 2.6 to 10% of all fractures in adults [1, 2]. The treatment of displaced midshaft clavicle fractures are a point of on-going debate. It is especially unclear whether operative management leads to better long-term functional outcomes [3]. Recent studies do show superior patient satisfaction after operative management [4, 5]. It is unknown which factors contribute to the difference in patient-reported treatment satisfaction between groups who experienced operative and nonoperative treatment.

Qualitative research can be helpful in exploring complex phenomena observed in research [6]. One such approach concerns the use of focus groups. This technique is characterised by group interaction that enables to structurally elicit opinions and preferences, explicitly aiming to generate data. Participants are able to build on each other’s statements, react to comments, pose clarifying questions, corroborate shared experiences and/or add divergent points of view [7]. Focus groups are often used in customer satisfaction studies to define the concept of satisfaction, identify the ingredients of satisfaction, and discover the conditions or circumstances that influence satisfaction [8]. To date, no focus group studies have explored treatment experiences after treatment of displaced midshaft clavicle fractures.

The aim of this focus group study was to explore and identify contributors to overall patient satisfaction after operative and nonoperative treatment for displaced midshaft clavicle fractures in adults.

## Materials and methods

### Design

**Fig. 1:**
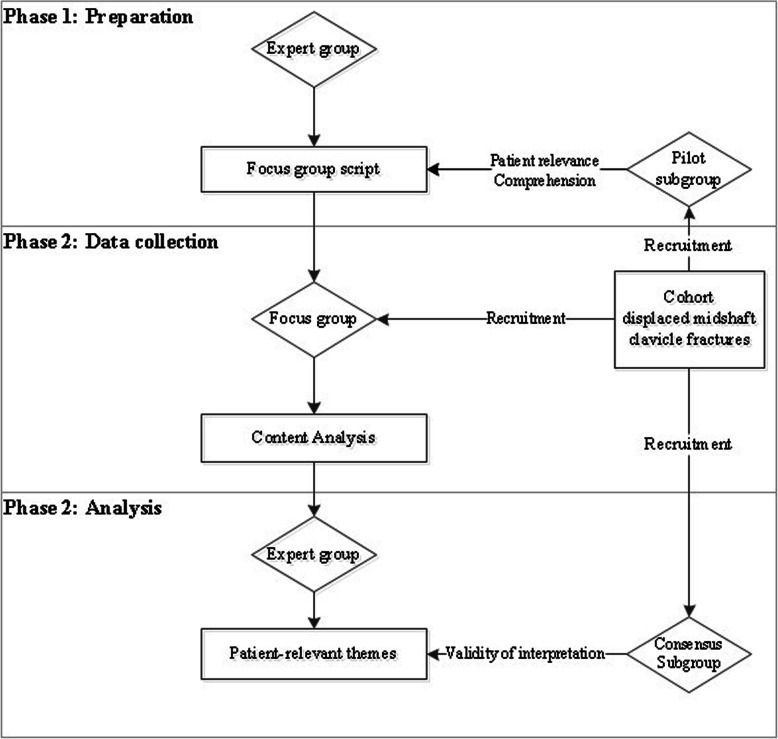
Flow chart study design

A focus group study was conducted. The study consisted of three phases: preparation, data gathering and data collection (Fig. 1). The medical ethics committee of the University Medical Center Groningen provided a waiver for the study (METc 2017/186). Written consent was obtained at the start of the focus group. All patient data and responses were coded before analysis.

### Phase 1: preparation

#### Focus group script

A focus group script was developed by an expert group consisting of two medical doctors with experience treating clavicle fractures and two methodologists. The first part of the script involved open-ended questions, facilitating free discussion. The topic of satisfaction was addressed by asking about pre-existing expectations, attitudes towards receiving other treatments, and perception of different dimensions of care received. The second part of the script was based on the conceptualisation of quality in health care as proposed by Donabedian [9]. According to this model, healthcare quality is evaluated in terms of structure (context of the healthcare delivery), process (clinical processes followed in healthcare delivery) and outcome (effect on the patient following healthcare delivery). This model has been previously used to evaluate trauma care [10, 11]. Additional hypotheses and logic for the formulated questions are shown in Table 1. After piloting the script in a subgroup of 10 patients currently suffering from displaced midshaft clavicle injuries, no major adaptations to the script were deemed necessary. The script was evaluated after each focus group. No adaptations to the initial script were deemed necessary based on participants’ feedback.

**Table 1:** Script, question format and logic

	No.	Question	Question logic
**Free discussion**	1	What did you expect of the treatment for your broken collar bone?	Contrasting expectations to actual level of care
	2	Looking back at your own treatment, how satisfied are you with the treatment? -What went well? -What might need to be improved?	
	3	Would you accept having the other treatment (e.g. sling instead of surgery)?	Compare beliefs for the other treatment to actual level of care
**Facilitator-guided discussion**	4	What did you think of the treatment at the E&A department?	*Dimension: Process of care* Hypothesis: no difference between treatment groups
	5	What did you think of your visits to the outpatient clinic? -Involvement in treatment decision -Guidance throughout process	*Dimension: Process of care* Hypothesis: interpersonal factors important
	6	What do you think of the result of the treatment? -Recovery of function -Pain -Cosmetic result -Complications	*Dimension: Outcome of care*
	7	What do you think of the facilities where the treatment was given? -Travel time -Waiting times	*Dimension: Structure of care* Hypothetical waiting times important to overall satisfaction
**Summary**	8	Are there any relevant topics missing in our summary?	Data saturation

#### Treatment groups

A chart review was performed to obtain data on patient’s treatments, duration of follow up and complications. No patient reported outcomes or function or objective functional measurements were available for this study.

Patients were classified into either nonoperative or operative treatment groups. Nonoperative treatment was described as treatment with a sling or collar-and-cuff. Patients who underwent delayed surgery after initial nonoperative treatment for a minimum of 4 weeks were recorded as nonoperatively-treated patients analogously to an intention-to-treat principle. Operative treatment was described as operative reduction and internal fixation (ORIF) within 4 weeks of injury. All operations were performed using locking plates. A four-week window was chosen to be inclusive of patients with a delayed presentation to the outpatient fracture clinic, followed by scheduling for elective surgery. All patients were treated by dedicated orthopaedic trauma surgeons.

### Phase 2: data collection

#### Participants

All participants were recruited from two community hospitals in the Northern Netherlands. Eligible patients were aged 18–65 at time of presentation, had sustained a unilateral displaced midshaft clavicle fracture Robinson 2B1 or 2B2 [2], presented to one of two participating centres between 2015 and 2017, and spoke Dutch. A purposeful sampling strategy was used to obtain variation in gender, age, treatment complications and outcomes [8]. Eligible patients were contacted by telephone and asked to participate in the focus group corresponding to their treatment. Upon completion of the focus group all participants were rewarded with a gift voucher of 20 euros.

#### Data collection

Data was collected through four face-to-face and two online focus groups. The latter was included in the design to facilitate participants who were unable to attend in person. Separate focus groups were hosted for nonoperative and operative treatment, as it was assumed that high homogeneity among participants in a focus group increases group dynamics and yields higher-quality data [8]. We aimed to host between two and four focus groups per treatment group, depending on data saturation reached. Data saturation is the point at which no more information is extracted from subsequent focus groups [8]. A minimum of 3–6 participants were invited per group. All focus groups were audio-recorded in a quiet room. Focus groups lasted 90 min with a short coffee break. The facilitator (OD) of the focus group had experience in orthopaedics and traumatology, but was not involved in the treatment of the participants. An observer (JS) was present to take notes and handle the audio recorder. Online focus groups were held by having participants log in to a password-protected website using anonymous credentials. Participants were asked to respond to questions posted by the moderator based on the research script. The online forum was kept open until no new contributions had been posted for three consecutive days, thereby assuming data saturation. Before publication, all patient quotes were translated by a native English speaker.

### Phase 3

#### Data analysis

Data was analysed thematically using an inductive approach [12]. All focus groups recordings were transcribed verbatim. A report was written for each of the individual focus groups in a question-by-question format with amplifying quotes. Two researchers (ET, OD) analysed the data separately, assigning codes to parts of text describing the meaning. Codes were then grouped into themes. The two lead investigators held a series of meetings to review the emergent themes, ensuring agreement. Themes were interpreted, compared and contrasted between focus groups. Coding was performed digitally using the qualitative research software package ATLAS.ti 8.0 [13]. After analysis, participants from the focus groups were invited to comment on the analysis to ensure completeness of analysis.

## Results

### Sample description

A total of 104 eligible patients were identified; 33 of them could not be reached and 24 agreed to participate (14 in the nonoperative group, 10 in the operative group). Three from each group participated in the online focus group. Participants from both groups were comparable in terms of age, time after treatment, and number and types of complications experienced (Table 2). Participants in the operative groups had more fractures on their dominant side. No pre-existing medical conditions were apparent in this group compared to two patients who had comorbid disease in the nonoperative group.

**Table 2:** Participants’ characteristics

	Nonoperative (***n*** = 14)	Operative (***n*** = 10)
**Age (years)**	45.8 years	40.7 years
**Male gender**	11 (78%)	6 (60%)
**Relevant comorbidities** **(e.g. malignancy, cardiovascular disease, pulmonary disease)**	2 (14%) - aortic aneurism - COPD	None
**Employment** **physical labour (%)**	4 (28%)	6 (60%)
**Fracture dominant side (%)**	1 (7%)	4 (40%)
**Length of time since injury (days)**	574 days	545 days
**Operative treatment (%)**	2 (14%)*	10 (100%)
**Full return to function (%)**	12 (86%)	8 (80%)
**Current pain levels > 3/10 (%)**	4/10	2/10
**Overall satisfaction with treatment (NRS)**	6.5/10	7/10
**Any complication (%)**	7 (50%)	4 (40%)
• **Type of complication**	Frozen shoulder 4x impaired union (delayed union or nonunion)	Frozen shoulder Sensibility loss Brachial plexus irritation Delayed union

### Analysis of qualitative data

Identical themes contributed to patient satisfaction in both treatment groups. A general lack of information was the most important factor of discontent. Three other main themes were functional recovery, speed of recovery and doctor-patient interaction (Table 3). After analysis, participants from the focus groups confirmed that our presented themes were the most important factors contributing to satisfaction after treatment. All factors will be described. Patient quotes are preceded with a code, formed by patient’s age at presentation, gender (M = male, F = female) and treatment group (O = operative, *N* = nonoperative).

**Table 3:** Main themes and subthemes

	Themes	Subthemes
**Main themes**	Information	Pros and cons of treatment options Being heard X-ray explanation Rehabilitation advise
	Recovery from injury	Range of motion Strength Return to function Pain Irritation from surgical hardware Cosmetic result
	Speed of recovery	Speed of diagnosis Delay to surgery Duration of follow-up Time to return to function
	Doctor-patient interaction	Empathy Trust Kindness
**Minor themes**	Parking facilities Travel distance/time Waiting times at the outpatient department	

### Information

The treatment decision was a key phase in the treatment process for many participants. Participants expressed a need for thorough understanding of the treatment options and implications. Participants were looking for certainty in choosing the best treatment approach. Several participants said they delegated to their surgeon, who was the expert in making the best decision. Others indicated wanting clear-cut information on percentages of risks and benefits, and were dissatisfied when this information was not provided to them.

*25M-O: The surgeon gave a confident and competent impression, and the pros and cons were discussed […] what I could expect and how long it would be before I could do everything again was clear [… ] we made a joint decision to have the surgery, and I’ve never regretted it.*

*9M-N: I understand that I’m responsible for my own choices […] let’s just look at percentages: 60 percent have it, it’s 40, most are in that corner, and I choose that too […]. I think, just give me the information, I’m asking for it*

Many patients turned to X-rays or other imaging modalities for certainty or confirmation in the treatment decision. Participants expressed that they found an explanation of the imaging to be important, and were disappointed when no images were shown.

*61F-N: I didn’t see any X-rays either, they didn’t show me what the fracture looked like, so I’ve been making a fuss about it.*

Some even requested the X-rays to ascertain a progression of consolidation.

*50F-N: […] when I asked a day later for the X-ray to see if that was really the case […] is it really attached.*

Preferences for treatment were also very much based on the radiological findings. Some participants thought that nonoperative treatment can only result in good outcomes if the X-ray shows bony apposition.

*43M-N: If conservative treatment gives good results, I wouldn’t really have to or want to be operated, as long as they could clearly confirm whether the sling would be enough […] if the parts of the collarbone touch against it.*

Likewise, participants found it especially difficult to accept nonoperative management when they considered the fracture to be severe.

*49M-O […] if you see that it’s split and is broken and split in three places, and you think to yourself, you can see even as a layperson that it is not going to heal by itself.*

*22M-N: I myself don’t understand how it can be expected to turn out all right if you have multiple fractures and the bones are out of place.*

Strikingly, several patients in the nonoperative group did not regard a sling or collar-and-cuff as a treatment at all – rather, they felt dismissed and felt like nothing was being done about the fracture.

*50F-N: They told me what I had and didn’t do anything more with it […] to me that’s not a real treatment.*

Delayed union after nonoperative management was common and often resulted in surgery. Although initially patients were relieved that they did not need surgery, choosing nonoperative management and switching to the operative group caused a feeling of regret.

*37M-N 3: Well maybe at that moment you like the idea of not having an operation […] Later on you will see it differently, but at that moment you think it is the better option.*

More information was also needed regarding rehabilitation after injury. Some would have liked advice on how to perform daily activities in the acute phase, others stated that they would have liked focused exercises or a referral to a physical therapist to aid their recovery.

*22M-N: You have no tips on how to climb out of bed or anything like that […] You don’t know that, nobody tells you.*

*49M-O: […] You’re on your own, make an appointment with the physiotherapist, who will help you. Then you do some research on the Internet and then, then you figure it out, but I mean, in the end you have to sort it out yourself.*

Likewise, all participants who did receive a referral to a physical therapist reported a benefit to their perceived recovery.

*60M-O: Yes, indeed with physiotherapy […] If you do the home exercises, because you don’t have that much to do anyway, you do improve really quickly.*

### Recovery from injury

#### Functional recovery

All participants expected full recovery in terms of strength and range of motion. Although many did recover, others experienced continuing disability, with some unable to resume work, sports and other hobbies.

*60M-O: I expected to recover fully. I had assumed that. A year-and-a-half and two operations further, yes it still bothers me […] I also can’t do the work I used to anymore, I can’t do my hobbies.*

*49M-N: And actually it took about two weeks, I could do almost everything again, I could do it. If you think about it, it’s fast […] Imagine, two weeks and it’s 100 percent good again. But it never recovered completely […]*

#### Pain relief

Initial pain relief was important in determining satisfaction. Especially participants experiencing delayed union reported prolonged periods of pain. However, patients from both treatment groups reported continued discomfort when wearing a bra, seat belt or backpack even after bony union.

*54M-N: That pain, you see […]. I can do everything again. But if I stay a bit longer in one position then it does become painful. The pain is a 3 or 4 on a scale of 10, I think.*

*31F-N: Sometimes it pulls to the shoulder, but also when I have something around it, like the car seat belt, it feels piercing, as if someone had stuck a knife in it. I feel it for a moment and then it’s gone […] a handbag, that is also uncomfortable.*

#### Cosmetic result

Aesthetic appearance, including scars, bumps, asymmetry and posture, was a minor issue for participants from both groups as long as they recovered functionally.

*61F-N: I’m very satisfied, I can do everything again. Only that bulge doesn’t look that nice, but okay.*

*38M-O: yeah, beautiful is something else. You can see the scar tissue but it doesn’t bother me.*

### Speed of recovery

Most participants expected a quick recovery and were disappointed when their actual pace of recovery did not match this expectation.

*33M-O: I thought they would put a plate, then take it out. Done. But things went differently. I thought that with all the advances nowadays, they operate and you’re done with it, […] everything will work like it used to. But it isn’t working.*

Especially participants in the nonoperative group had negative associations with treatment, as they felt it took too long.

*28M-O: What gets me is that first they said that it can heal naturally and then they said nah we’ll have to operate. Thinking back, I probably would’ve preferred them to put a plate there immediately, because now I am ten or eleven weeks further.*

The feeling of decisional regret was reinforced by a feeling of seemingly quick recovery after surgery.

*24M-N: One day after the operation I could do as much as nine weeks without the operation.*

Especially semi-professional athletes had a clear preference for operative management, based on experiences among team members, friends or relatives of faster return to function.

*29F-O: I made a rather quick decision to let them operate, even though it was probably a no-brainer, as in other people’s experiences it heals more quickly.*

One participant experiencing delayed union would have liked earlier advanced imaging (MRI, CT-scan) to have extra certainty about choosing the right treatment.

*43M-N: Maybe when in doubt […] do an MRI immediately anyway […] so they can assess more quickly whether a sling is the first choice or they should operate.*

Even patients from the operative group indicated feeling disappointed with the delay to surgery.

*55M-O: I am quite satisfied with the treatment, only the operation should have been done sooner than two weeks after the accident […] those are two lost weeks.*

### Doctor-patient interaction

Patients across treatment groups were satisfied with the interaction with their treating surgeon. Surgeons were generally regarded as trustworthy and sympathetic. Some participants did feel that their surgeon was rushing, thereby negatively influencing their treatment experience.

*38M-O: The first doctor that treated me was rather distant and didn’t seem to have a lot of time. The second doctor was exceptionally good and really took the time.*

### Other minor items

#### Emergency department

All patients perceived their initial treatment at the emergency department as satisfactory. This was expected, as in our practice patients presenting to the emergency department with a clavicle fracture are routinely referred to the outpatient clinic for definitive treatment decisions.

### Waiting times, travel distance, hospital interior

Our script touched on other items like waiting times, travel distance to the clinic and hospital interior. None of these were mentioned by participants in the free discussion, and were thus regarded as less important in determining satisfaction.

## Discussion

The focus group analysis identified four main themes for patient satisfaction: need for information, functional recovery, speed of recovery and doctor-patient interaction. Overall emerging themes were identical for both treatment groups, indicating that similar dimensions of care contribute to the patient experience irrespectively of type of treatment given.

Need for information was undoubtedly the most important emerging theme for participants from both treatment groups. Participants expressed wanting to be continuously informed about the injury, diagnostic imaging, treatment options and rehabilitation methods. These findings are similar to those of a recent qualitative study on patient experiences after trauma [14]. A lack of information was thus regarded as a major cause of discontent, in line with a similar study on patients’ expectations of orthopaedic consultations [15].

Most participants expected full recovery and fast return to function. This was previously also observed in another study on patients’ expectations in orthopaedic practice, in which participants wish for a quick fix to their problem [16]. Not surprisingly, continued impaired strength, limited range of motion and pain had a negative effect on overall patient satisfaction in this study; this was true for both nonoperatively and operatively treated patients. The timing of treatment and return to function also contributed to dissatisfaction. Especially in the nonoperative group, longer return to function negatively influenced the overall patient experience and contributed to decisional regret for choosing nonoperative management. Interestingly, even patients who had surgery within the four-week time window defined as operative management complained about the delay to treatment, which indicates a general desire for a quick resolution of symptoms across treatment groups. This notion aligns with another focus group study in orthopaedics, where participants preferred outpatient surgery and placed it within the wider societal context of efficiency and speed. They wanted surgery to be like a fast-food experience, with its emphasis on speed, predictability and control [17]. Along the same line of thought, several patients in this study regretted not having advanced imaging (CT, MRI), as in their opinion this could have prevented delay to surgery.

Crucially, nonoperative management with collar-and-cuff or sling was not regarded as a proper treatment by several participants in the nonoperative group. Patients felt they were not taken seriously and felt dismissed by their surgeon. We observed the same finding in a retrospective study among a different cohort of patients [4]. An explanation for this phenomenon can be that patients with pain, deformity and striking radiographic findings might imagine that they have no other option than operative fixation [18]. This finding warrants careful consideration and explanation of the reasoning for choosing nonoperative treatment by surgeons treating clavicle fractures.

Doctor-patient factors like trust, compassion and empathy did not dominate stakeholders’ perspectives of patient satisfaction, as was the case in another study exploring patient satisfaction in orthopaedic practice [19]. Patients may be less geared towards empathy and more towards functional recovery after a relatively minor injury like a clavicle fracture. In contrast to previous studies, travel distance, waiting time and care facilities were not mentioned spontaneously and should therefore be regarded as unimportant to overall satisfaction in our cohort. A likely cause for this notion is the vicinity of local hospitals and the few follow-ups needed for the treatment of clavicle fractures.

Satisfaction is often defined as a measure of how products and services meet or surpass expectations [20]. Dissatisfaction arises from the inability to meet pre-existing expectations [21]. Expectations can be unmet either because of poor recovery from injury or because the expectations itself are unrealistic. A recent systematic review shows that both nonoperative and operative management may result in good functional outcomes [22]. In a cohort similar to the present study we did not observe a functional difference between the treatment groups either, nor did functional outcomes predict satisfaction [4]. Our results therefore suggest that discontent likely arises from a failure to address unrealistic expectations.

Based on our findings and the current literature, we believe that either treatment strategy yields satisfactory outcomes. However, we stress the need for orthopaedic surgeons to explore patients’ needs and expectations so as to enable an optimal treatment decision, and when necessary correct unrealistic expectations to prevent dissatisfaction. Information and guidance should continue throughout the treatment to instil confidence and address the extent of recovery patients can expect, the expected time to recovery, and how and when to use the injured limb in order to gauge their progress and be practical when planning their lives [14].

To the best of our knowledge this is the first study to detail views of patients after treatment of displaced midshaft clavicle fractures in adults through a qualitative-based approach. The main strength is that both nonoperatively and operatively managed patients were invited to participate. We also choose to include an online focus group in addition to the face-to-face groups to facilitate participants who were unable to attend in person. We believe that this is justifiable, as previous research shows that online bulletin boards yield similar qualitative data as face-to-face groups [23] and the same was observed in our study. Our script was carefully developed by a team of clinicians and methodological experts, and piloted in patients with clavicle injuries. The script tapped into the concept of satisfaction using different approaches (expectations, attitudes and perceived care), yielding conceptual density [24]. Hardly any new insights were provided in the facilitator-guided discussion and no major issues were overlooked based on participant feedback, which indicates a robust script. Additional focus groups provided less new information, indicating adequate data saturation. The number of former patients in this study also corresponds to similar studies. For example, a review of 15 focus groups on knee osteoarthritis reported a median of 20 subjects per study [6]. It is therefore unlikely that additional focus groups would have provided more information or different results.

There are limitations to the design. First, focus group research relies on memory of past events, experiences and conversations [8]. The effect of recall bias is probably small as all participants sustained a clavicle fracture within 2 years before the focus group, and none of the participants stated difficulties recollecting their experiences. Next, analysis of results is open to discussion as it is based on subjective coding. We tried to maximise the robustness of our analysis by involving patients in the design and interpretation of the study results, and by having two analysts handle the data independently from each other. Future research should prospectively study the effect of improved shared decision making on overall patient satisfaction and the relevance of patient satisfaction as an outcome in clinical research.

## Conclusion

Our study shows that informing patients about their injury, treatment options and expectations for recovery is paramount for overall patient satisfaction after treatment for a displaced midshaft clavicle fracture.

## Data Availability

The datasets used and/or analysed during the current study are available from the corresponding author on reasonable request.
